# An Address Lately Delivered to the Medical Society of Horncastle, on Endemic Causes of Disease

**Published:** 1802-09-01

**Authors:** C. Harrison


*An Address lately delivered to the Medical Society of Horncastle, on Endemic Causes of Disease; by* C. HARRISON, M. D. *by whom it has been communicated to the Editors.*

GENTLEMEN,

I Riſe to thank you for ſelecting me for your Preſident.—To be choſen the firſt among equals, by the unanimous ſuffrages of independent Aſſociates, is, in my eſtimation, the higheſt honour that can be conferred upon any individual. Impreſſed with this ſentiment, I beg leave to acknowledge your partiality towards me in terms of the warmeſt gratitude, and to aſſure you that, on my part, no exertion ſhall be wanting to promote the many advantages which may be derived from our Society: theſe I will take ſome future opportunity to enumerate and explain; but at this time I muſt neceſſarily paſs over ſeveral of them without notice.

The county of Lincoln is, as you know, of great extent, and contains a variety of ſoils which appear to have no ſmall influence upon the health and the lives of its inhabitants. To diſtinguiſh theſe from each other, by chemical analyſis, and aſcertain the diſeaſes which depend upon them and other local cauſes, are objects of great national importance, and may be accompliſhed, in a great meaſure, by the combined efforts of all all our fellow members. To complete an undertaking of this extenſive and multifarious nature, requires the zealous co-operation of numerous individuals; and I make no doubt, as this county happily contains many patriotic and ingenious characters, if we deſire aſſiſtance, materials for this purpoſe will be induſtriouſly collected, and liberally communicated.

The town of Horncaſtle is in a central part of the county, and upon the edge of the Wolds. On both accounts it is well ſituated for the inquiry; and as the Society conſiſts of Members, who practiſe in various places, it will be leſs difficult to obtain the neceſſary information.

Dr. Mitchell, of New York, and other eminent Chemiſts and Philoſophers, entertain an opinion, that countries abounding with calcareous matters are ſeldom viſited by epidemic complaints. The extenſive range of elevated grounds, which conſtitute a great part of Lindſey, are called the Wolds of Lincolnſhire. Theſe, and the Cliff Row, which is ſituated chiefly in Keſteven, are entirely calcareous. The diviſion of Holland, with the marſhes, fens, and other low lands, are likewiſe of great extent. They conſiſt chiefly of ſandy, loamy moor and clay lands, which are, more or leſs, completely drained, and are in different degrees of cultivation. Theſe tracts either contain no calcareous ingredients, or the ſtrata are buried ſo deep in the bowels of the earth as not to affect the ſurface of the ground, or the incumbent atmoſphere. Since the county of Lincoln includes ſuch a variety of ſoils, I conceive, that by making diligent and minute inquiries every where among the aged and intelligent inhabitants; by collecting and comparing the parochial regiſters in all the market-towns and villages for the laſt fifty years; and by ſoliciting information from medical practitioners and literary men, we may obtain ſuch a knowledge of the epidemic and prevalent diſeaſes of the county as will enable us, in our reſpective ſtations, to be of greater ſervice to our patients, to our relations, and the community at large.

I have reſided at Horncaſtle upwards of fourteen years; and although I have been actively engaged, during that time, in the exerciſe of medicine, I do not recollect any diſorders except the influenza, ſmall-pox, meaſles, and hooping-cough, to have been epidemic in this place before laſt year, when a typhus fever was brought into it by ſome ſtrangers.

This fever has, I believe, been very general in other parts of the county, as well as at Horncaſtle; but whether it has prevailed more in calcareous or other diſtricts, are circumſtances upon which I can give you no information.

According to Dr. Mitchell and his followers, azote, or, as they they term it, fepton, is the caufe of moft epidemic, and of many fporadic diforders. It exifts naturally in the atmofphere, under the appellation of azotic gas. All putrefying animals, and many corrupting vegetables, fupply it copioufly; but fo great is its affinity to caloric, oxygen, and many other things, that it has never been obtained feparate and ifolated. He fuppofes fepton to favour the growth of vegetables, and to be of great fervice in agriculture. In a concentrated ftate, he thinks it deftroys vegetation by too powerful a ftimulus, and produces many very formidable and virulent diftempers. Septon, united to caloric and oxygen, forms a great variety of compounds, which are greedily attracted by calcareous fubftances, &c. Neutralized by this means, it exerts no deleterious effects upon the human conftitution. In calcareous countries, the excefs of fepton in the air is prevented by its attaching itfelf to the quicklime of the foil, as faft as it is extricated. In other places, it is wafted about in the atmofphere, and forms various compounds, by which its virulence is reftrained or modified.

According to this opinion, it follows, that calcareous regions are lefs expofed to epidemic maladies, and to feveral difeafes, which afflict the inhabitants of other places. I have reafon to believe, that the prevalent complaints upon the wolds and in the fens differ confiderably from each other. The confumptions upon the wolds, I have been accuftomed to impute to an oxygenated atmofphere, and the calculous affections to the prefence of calcareous impregnations in the wells and rivers. In the fens, idiophatic confumptions and ftone are feldom obferved; but in fpring and autumn, the inhabitants are expofed to obftinate agues and remitting diforders. Of late years the fens have been much more healthy; and when, by the works that are now conftructing, a complete drainage fhall be obtained, I think that they will become not only among the moft valuable agricultural tracts in the ifland, but among the moft falubrious to the inhabitants. By the deftruction of organic bodies, and in many other ways, this principle is extricated in all countries. In burying grounds and in flaughter houfes, &c. it is abundantly produced, and has been fuppofed by many phyficians to render the air unwholfome to the inhabitants. Before the time of Dr. M. feveral deftructive and wide fpreading epidemics had been traced to putrefying fubftances; and it becomes a matter of ferious inquiry, how far the cuftom of interring dead bodies in churches and in towns ought to be permitted, or ought to be interdicted, by the authority of government.

For my own part, I can fee no advantage that will accrue to individuals from perfifting in the practice, and therefore I fhall be happy to fee it difcontinued.

During

During the deftruction of animal matters, volatile fubftances certainly afcend from the ground and mix with the atmofphere. The more fixed principles are diffolved in the rain water that defcends into burying grounds, and, paffing with it through the bowels of the earth, are received into wells, from which the inhabitants of towns are fupplied with water for culinary purpofes. Should it turn out that the air and the water of fuch places are not peftilential, I do not fuppofe that the gallantry of Britons is fo truly romantic, as to receive any gratification from inhaling or drinking the exuviæ of their deceafed lovers or anceftors. By burying a long time in church-yards, the foil becomes raifed above the floor of the church; this makes the ground damp, and the moifture, with its various impregnations, afcends into the upper parts, and contaminates the whole air of the church. Many difeafes are occafioned by moifture acting upon the human conftitution; and as its influence is greatly favoured by reft, invalids are expofed to danger by remaining long in fuch fituations.

Without imputing too much to the effects of aqueous and atmofpherical impregnations, I fhould recommend that burials in towns, where practicable, be difcontinued, and the floors of all churches raifed above the furface of the adjacent grounds. Till this be done, and free ventilation encouraged, the devout inhabitants will be in danger of fuffering from the damp and ftagnant air in which they are enveloped, during the period of this neceffary and important part of their duty.

Evils of this kind may be eafily removed, or I fhould have had great reluctance in difturbing your minds, although in a matter of fuch importance to your health and comfort.

But the difeafes to which I am at prefent defirous to call your particular attention, are, pulmonary confumptions; calculous affections; and intermittent diforders. They are each of them fubjects of great importance, and from their frequent recurrence among us, are deferving of your moft attentive confideration.

I had not refided long in this divifion of Lincolnfhire, before I was ftrongly impreffed with an idea that the inhabitants upon the wolds were a great deal expofed to idiopathic confumptions, and peculiarly liable to calculous complaints. In the divifion of Holland, and the extenfive marfhes of our county, thefe diforders are probably lefs known than in moft *other situations* in England. Multiplied experience and numerous inquiries have tended to confirm thefe opinions; and fince the eftablifhment of our Difpenfary, I have been collecting facts to elucidate them. The interruptions to which medical practitioners are continually expofed muft plead an apology for laying my my ideas before you in a crude and imperfect form. Should my fentiments be fortunate enough to meet your approbation, I fhall return to the inquiry with frefh ardor, from a conviction thereby afforded that my obfervations will be ufeful to fociety.

Phthifical complaints are the great fcourge of this ifland. Pulmonary confumptions alone are reported annually to deftroy upwards of twenty thoufand perfons.

How many of our countrymen are carried off by the other forms of hectical diforders, I am unable to calculate; but their numbers are certainly very great. I would not be underftood to affert that phthifical complaints of all kinds are more prevalent upon the Wolds. My obfervations have been chiefly directed to affections, which originate in fcrofula, and thefe I am inclined to believe are much lefs frequent in the divifion of Holland, than that of Lindfey. When it is confidered that the victims to fcrofulous affections are chiefly the fair and the delicate, who have arrived at an age to be ufeful to their friends, and promifed by fuperior mental endowments to be a bleffing to the community, we cannot too much deplore and lament thefe premature deaths.

The fituation of this town has afforded me numerous opportuniteis to inveftigate more particularly pulmonary complaints, and I can truly affert that they are much lefs frequent in the fens and marfhes than in other parts of my circuit. The difference with refpect to idiopathic confumption is very great indeed: In fome parts where I practife, it is a very common complaint, and in others it is fcarcely known to the faculty. Pulmonary confumptions are certainly to be met with every where; but when I have been confulted upon fuch cafes in our marfhes, or the divifion of Holland, I could either trace them to other fituations, to neglected colds, or fome irregularity in the fuffering perfon.

Confumptions from thefe caufes are occafioned by accidents to which all perfons are expofed in their commerce with the world. It is not to them that I refer; it is to the florid and tubercular confumptions alone that my obfervations are directed. Thefe are fo much the effects of predifpofition, and are fo infidious in their attacks, that it is very difficult, if not impoffible, to prevail upon invalids to adopt precautionary regulations fufficiently early to prevent their deftructive and fatal confequences. Arguments are infufficient to alarm the phthifical, and make him alive to his dangerous fituation. The mind is too confident to be moved by admonition. Nay, when the complaint is fully eftablifhed, and has made confiderable progrefs, the patient ftill continues free from apprehenfions, and cannot be

 made made to perceive the unavoidable mortality which awaits him. In thefe melancholy cafes, there is no fafety but in flight. The fufferer muft either be removed into another air, or little good can be expected. Happily, the approach of the difeafe may commonly be diftinguifhed while the conftitution is fo little injured that a cure may be reafonably anticipated. This in my opinion will never be accomplifhed by enclofing patients in rooms filled with medicated air, or a reduced atmofphere. The effects of fuch difcipline are too tranfient to produce any permanent change upon difeafed lungs. When immerfion is finifhed, and the patient returns again to a common atmofphere, I fhould even be difpofed to apprehend bad confequences from the fudden tranfition. If we can fortunately difcover any dif-trict, where idiopathic confumptions are entirely unknown, we may expect that fuch patients will derive great benefit from removing to it, and refiding there, till their fufferings be re-moved, and good health completely reftored.

When I firft took up the opinion, that pulmonary confump-tions were feldom produced in the divifion of Holland, I ap-plied for information, among others, to Mr. Wayet, an old and experienced practitioner at Bofton, who informed me that pulmonary complaints of all kinds were very uncommon in his neighbourhood, and that fuch as did occur were milder than in other places. In an extenfive practice of more than forty years, he had feldom attended in cafes of peripneumony, or fpafmodic afthma; and meafles, he obferves, were feldom dangerous in that part of the country.

This opinion of Mr. Wayet is agreeable to my own expe-rience, and that of feveral medical practitioners with whom I have converfed upon this interefting fubject. I accidentally mentioned it, a few years fince, in the company of Mr. Bouche-rett, Member of Parliament for Great Grimfby, a gentleman of unbounded candor, and a moft benevolent difpofition. He was fo much ftruck with the remark, that he took an opportu-nity to make inquiries concerning it among his Dutch friends, and was informed that confumptive diforders feldom occurred among them. From the great refemblance of the foil and air in the Batavian republic and the Lincolnfhire fens, I am dif-pofed to believe, that the exemption from phthifical complaints, in both countries, depend upon the fame caufes. In the lower parts of this county the air is moift and foft, when compared with the atmofphere upon the Wolds; the lungs are there-fore lefs irritated in it, and in confequence, as I conceive, tu-bercles, which lay the foundation of idiopathic confumptions in other places, are feldom met with in thefe fituations.

Sheep, it is well known, are liable to be affected with tubercles cles in the lungs, and to die hectical. By what morbid action tubercles are formed in thefe animals, and whether they remain indolent for fome time, or are roufed into immediate action, are fubjects upon which I can give no fatisfactory information. In human lungs, the fuppurative procefs is often very flow. Unlefs the lungs be hurried into fuppuration by mifmanagement or irregularities, they remain ftationary for feveral years, and, under favorable circumftances, appear to be gradually abforbed. In every cafe where the exiftence of tubercles is fufpected, I fhould recommend the patient to remove into a foft moift atmofphere, where the lungs will be lefs ftimulated; and to remain in it till he has reafon to believe that the tubercles are entirely removed. Whether more eligible places are to be found in other parts of the kingdom, I am unable to determine; but till we have obtained better information upon the fubject, I am difpofed to recommend a refidence in the Lincolnfhire fens to fuch patients, in preference to every fituation with which I am acquainted.

By making experiments upon fheep in different diftricts, fituations and circumftances, I am difpofed to believe that fome comparative knowledge would be obtained, which might affift our inquiries into the prevention and cure of tubercular confumptions in the human body. If, for example, a flock of fheep were to be bred, and wholly maintained upon the Wolds till they were fit for the butcher, and another were to be kept in the fens, an opportunity would be given to examine the lungs in each, and to determine how far the different flocks were affected by their refpective pafturage. Such an inquiry would probably enable us to treat the difeafes of fheep with more fuccefs, and thus a double benefit be conferred upon fociety by the inveftigation.

In January, 1793, a young lady, aged about twenty, who had been my patient feveral months, with very ftrong fymptoms of florid confumption, went upon a vifit into the neighbourhood of Wifbeach, in the Ifle of Ely. While under my care fhe had continual pains in the cheft, with a fhort dry cough, the fputum being generally ftreaked with blood. Motion foon fatigued her, and increafed the dyfpnœa and cough; fhe evidently loft flefh and ftrength, and in an afternoon and evening fhe had a flight hectical fit. Her being of a confumptive family, I confidered as affording a *prognostic* unfavourable to her recovery. After an abfence of three months, fhe returned again fo much improved in health, that fhe thought herfelf quite well. She took up her refidence at an elevated village in this neighbourhood. I frequently faw her, and had the mortification to obferve an infidious attack of all her old fymptoms. Before the end of the fummer, I thought her as bad as ever fhe had been, and urged her to return to her friends near Wifbeach. With this advice fhe neglected to comply for two years, during which time her fymptoms rather increafed than abated. After a fecond vifit of near four months, in the winter and fpring 1795, fhe returned again to her relations, where fhe continued feveral years without fuffering much from phthifical complaints, though her conftitution remained infirm, and her complexion was pale and fallow. About two years ago fhe married, and now refides in a diftant county, where fhe enjoys better health, and is become a mother. I have been told by a medical acquaintance, that an apothecary's wife was feveral times fnatched from impending confumption, by removing from Lynn Regis to Wifbeach. Her relief was fo ftriking and immediate, that before fhe had travelled three miles, fhe always found herfelf better, and in a few days her pectoral diforder entirely left her. Senfible of the great benefit fhe received from *this particular change* of air, fhe had recourfe to it, whenever affaulted by her phthifical fymptoms.

Should my opinion be confirmed, that idiopathic confumption is a very uncommon difeafe in our fens and marfhes, it will be a matter of great confequence to inveftigate the caufe of this exemption, and determine how far it ought to be imputed to the atmofphere alone, or to it in conjunction with other powers. It is a fubject of vaft importance, and will, I hope, receive the attention that it deferves. For my own part, I fhall be thankful for every communication, which has any tendency to confirm or overturn the theory that I have propofed, as it is my intention, at fome future period, to enter more fully upon the inquiry.

Permit me, before I conclude this Addrefs, to congratulate you upon the repeal of the Income Tax, a tax which however neceffary, certainly preffed more upon medical men than many other claffes of the community. Inconfiderate people may imagine, that in contributing for our profeffional receipts, we paid for no more than our profits and gains. A little reflexion will fatisfy the candid and unprejudiced, that of late years, efpecially horfes and fervants, independent of the taxes upon them, could not be maintained for a trifling fum; and yet it is impoffible to conduct a very moderate bufinefs in many fituations without them. Thefe, with other mifcellaneous expences, when added together, amount annually to no fmall fum, and are fo unavoidably connected with medical practice, that without incurring them, the emoluments could not have been at all obtained. Their previous fubftraction was therefore abfolutely neceffary to to bring the receipts themfelves under the denomination of profits and gains.

Income, in its ufual acceptation, is a loofe and vague term; it applies equally to grofs receipts and to net produce: But when the Legiflature had limited it to be fynonimous with profits and gains, it became as clear and precife as any other word in the Englifh language. By fuch a definition, it was reftricted in the act, as I conceive, to mean only net produce, or the fum that remains to profeffional men, after their neceffary and unavoidable expences had been previoufly fubtracted.

In addition to thefe I might ftate, that the Faculty are expofed to fo many cafualties which do not affect other employments, that no receipts are more precarious than ours, or require to be eftimated with more delicacy. A fit of ficknefs, to which, in the exercife of our profeffion, we are peculiarly expofed; any accident that produces confinement, or the unexpected termination of fome particular cafe, are each of them fufficient to curtail the emoluments of the moft able and popular practitioner.

Any obfervations upon a repealed act of parliament may be efteemed fuperfluous and irrevelant; but as there is reafon to believe that a tax upon income will at fome time be among the refources for a new war, I thought a few remarks upon the late bill might not be inapplicable to the prefent occafion, or foreign to the bufinefs of this day.

I have thus delivered to you my fentiments without any referve. I had likewife intended at this meeting to offer fome remarks about calculous and intermittent diforders. Thefe, however, I am obliged to defer till another opportunity; and in the mean time I fhall be happy to receive any communications about them, or the other fubjects concerning which I have prefumed to addrefs you.

